# Author Correction: Viewing life without labels under optical microscopes

**DOI:** 10.1038/s42003-024-06977-x

**Published:** 2024-10-04

**Authors:** Biswajoy Ghosh, Krishna Agarwal

**Affiliations:** UiT - The Arctic University of Norway, Tromsø, Norway

**Correction to:** *Communications Biology* 10.1038/s42003-023-04934-8, published online 25 May 2023

In this article, the funding from H2020 Future and Emerging Technologies Project ID 964800 was omitted in the funding section. The original article has been corrected.

